# Editorial: Generative AI and large language models in medicine: applications, challenges, and opportunities

**DOI:** 10.3389/fdgth.2026.1986903

**Published:** 2026-09-17

**Authors:** Fahim Sufi, Seedahmed S. Mahmoud

**Affiliations:** 1 COEUS Institute, New Market, VA, United States; 2 Centre for Trade and Investment (CTI), University of Dhaka, Dhaka, Bangladesh; 3 Shantou University, Shantou, China

**Keywords:** AI governance, clinical decision support, clinical validation, digital health, generative artificial intelligence, health communication, human AI collaboration, large language models

## Introduction

Generative artificial intelligence has entered medicine through a heterogeneous set of uses rather than through a single technological pathway. Large language models now draft clinical text, interpret reports, answer patient questions, support knowledge retrieval, structure health data, assist education, and participate in increasingly complex decision support workflows. The 34 contributions assembled in this Research Topic capture that breadth across clinical medicine, public health, digital health, oncology, rehabilitation, mental health, nursing, health informatics, education, quality management, and biomedical policy. Their collective value lies less in any single performance estimate than in the opportunity to examine what changes when language model research moves from demonstrations of linguistic competence toward systems that must operate under clinical constraints.

A consistent pattern runs through the Research Topic. Model capability has advanced more rapidly than the evidence needed to justify routine clinical reliance. Artsi et al. identified only four empirical studies meeting strict criteria for real world implementation through April 2025, despite a much larger literature based on retrospective or simulated evaluations. Those implementations suggested gains in efficiency and workload, but they also exposed problems of generalizability, regulatory delay, performance variability, and limited post deployment monitoring. Wang et al. similarly distinguish benchmark performance from clinical infrastructure, arguing that hallucination, privacy, validation, and governance remain unresolved even as general and specialist models improve. This tension provides the organizing argument of the Research Topic: the principal scientific problem is shifting from whether a model can produce a medically plausible answer to whether a complete clinical AI system can produce dependable, traceable, comprehensible, and accountable outputs under conditions that resemble actual care.

### Overview of the research topic

Across the collection, empirical model evaluations, clinical and public health studies, professional surveys, multimodal investigations, methodological frameworks, and legal or regulatory analyses examine distinct dimensions of medical generative AI. Their endpoints extend beyond accuracy to readability, actionability, reproducibility, expert agreement, workflow performance, traceability, and governance. This breadth makes the collection useful for assessing the transition from general model capability to task specific systems operating under healthcare constraints.

## Discussion of the contributing articles

### From benchmark competence to clinical usefulness

The studies concerned with diagnosis, staging, and clinical reasoning show why this distinction matters. In a two institution study of 552 PET CT reports across six cancers, Xu et al. found that GPT 5 achieved 82.1 percent overall TNM staging accuracy compared with 77.0 percent for junior radiologists, while repeated model agreement was close to perfect. Performance nevertheless differed across cancer types and was weakest for metastatic staging. Dinç et al. reported an analogous pattern in radiation oncology. GPT 5 reached 92.8 percent on a standardized examination benchmark, substantially exceeding earlier GPT generations, yet authentic clinical vignettes still produced substantive errors in cases requiring precise trial knowledge or detailed adaptation to clinical context. The high benchmark score therefore did not remove the need for specialist review.

Other studies reinforce the task dependent character of performance. Pan et al. found that DeepSeek V3.1, ChatGPT 5, and Grok 4 answered more than 90 percent of guideline derived bone cancer questions correctly in both evaluation rounds, although model specific strengths remained. In maternal venous thromboembolism care, Li et al. found superior overall expert ratings for GPT 4.1, Claude 3.7, and DeepSeek across education, care planning, and prevention, but explicitly did not interpret those results as evidence of readiness for independent nursing deployment. Konzmann et al., synthesizing 31 tumor board studies and 3,845 cases, identified a broader complexity gap: concordance was strongest in standardized and common oncological settings and deteriorated in rare, multimodal, or clinically ambiguous cases.

This evidence is compatible with the conceptual position advanced by Greengrass, who treats AI as a potential support for human clinical reasoning rather than as a replacement for it. Human reasoning is constrained by working memory, cognitive load, fatigue, anchoring, and other biases, whereas computational systems can search and compare information at scales that exceed unaided cognition. The collection, however, does not support the inference that computational scale removes clinical uncertainty. Its more defensible implication is a risk gradient. Language models appear most reliable when the task has explicit rules, sufficiently complete textual evidence, and an auditable reference standard. Their reliability becomes more difficult to establish as the task requires multimodal interpretation, rare disease knowledge, rapidly changing evidence, patient specific tradeoffs, or judgments for which several clinically defensible answers may exist.

### Grounding, system design, and bounded clinical tasks

A second theme concerns the architecture surrounding the language model. Several contributions suggest that the clinically relevant object is increasingly the full system rather than the foundation model in isolation. Barlow et al. fine-tuned an open Llama model to identify actionable incidental findings in PET CT reports and achieved a document level F1 score of 0.917 internally and 0.79 on an external test set. The reduction under external evaluation is itself informative, because differences in reporting style and institutional practice remain consequential even when internal performance is strong.

Grounding methods address a different source of error. Kokash et al. combined ontology-based retrieval with LLM semantic adjudication for healthcare data harmonization and reported expert agreement as high as 92 percent. Retrieval alone was insufficient; the gain came from pairing broad candidate retrieval with model based semantic validation. Chen et al. provide a related demonstration through a knowledge graph system for Jin San Zhen therapy. Their hybrid architecture outperformed the general model on correctness, professionalism, and completeness, while the knowledge enhanced outputs remained traceable to source literature and no fabricated claims were detected in the evaluated question set. Sugihara et al. extend this architectural reasoning to the choice between open and proprietary models. Proprietary systems may offer stronger schema adherence or complex reasoning in some tasks, whereas open models provide advantages in local control, auditability, customization, privacy, and cost. The practical choice therefore depends on the clinical task and governance environment rather than on a universal model ranking.

Bounded documentation and evidence processing tasks may be among the more defensible near term applications, but they still require verification. Aiumtrakul et al. found strong clinical reasoning in 89.7 percent of ChatGPT 5 generated nephrology prior authorization letters, yet diagnostic coding was correct in 79.3 percent and citation errors remained. Oami et al. observed high performance for background data extraction in systematic reviews, but outcome extraction was markedly more variable and repeated session consistency declined for the harder task. More elaborate prompting produced only modest accuracy changes while increasing processing time. Khoruzhaya et al. place these findings in methodological context: their review of medical text summarization reported external validation in only 7 percent of studies and patient safety assessment in only 3 percent, with wide variation in reported hallucination rates. Their MEDAI LLM SUMM checklist accordingly emphasizes clinical validity, prompt documentation, expert evaluation, hallucination assessment, safety, and data availability. Zhu et al., using eight models to generate medical examination questions and answers from real world EHRs, further show that models can approach clinicians on selected dimensions while human authors retain advantages in information sufficiency and overall answer quality.

Taken together, these studies distinguish prompt engineering from clinical system engineering. Prompt design can improve instructions, but evidence grounding, model adaptation, structured outputs, local deployment, source traceability, safety rules, and expert adjudication determine whether an apparently competent model can be integrated into a reproducible workflow.

### Patient communication, health literacy, and supportive care

Patient facing applications expose another limitation of accuracy centered evaluation. Graca et al. found that healthcare professionals rated ChatGPT responses more favourably than established evidence based PCOS responses for 11 of 12 questions, and further simplification improved readability. Yet the wider collection shows that medical correctness does not guarantee communicative usefulness. Joseph et al. found that cardiovascular responses changed in complexity with the education level implied by the prompt, but output frequently remained above ordinary health literacy needs. Wang et al. on gestational diabetes reported 92.17 percent mean accuracy for ChatGPT 5 on standardized questions and favourable reliability ratings, while all evaluated models still exceeded the recommended sixth grade reading level and showed poor transparency under JAMA criteria. Huang et al. reached a similar result for perinatal depression: responses were often informative and empathetic, but readability remained excessive and clinical safety behaviours were incomplete.

The chronic heart failure study by Wang et al. on chronic heart failure adds actionability to this evaluation framework. Overall content quality was favourable, but median actionability was only 20 percent, and the most complete output was also among the least readable. This reveals a structural tradeoff that cannot be captured by accuracy alone. Albaz et al. likewise found favourable clarity and tone in ChatGPT 5 responses about vasectomy, but medical accuracy and completeness varied across questions and expert agreement was very low. Patient validation was absent. Angyal et al. approach the same problem from the user side by developing a questionnaire to measure how adults use ChatGPT for primary and secondary disease prevention, providing a basis for studying behaviour rather than assuming that availability translates into appropriate use.

Two contributions show how patient and caregiver support may be made more constrained. Hanzawa et al. combined a domain specific knowledge base, retrieval augmented generation, sentiment analysis, explicit safety restrictions, and escalation rules in an agent supporting families implementing treatment for adolescent anorexia nervosa. Experts rated 91.6 percent of 477 responses as clinically appropriate, but the authors appropriately call for usability testing and randomized evaluation of caregiver burden, adherence, and clinical outcomes. O’Malley et al. similarly describe retrieval, system instructions, and fine tuning as complementary strategies for customized physical activity chatbots, while emphasizing human evaluation, privacy, and operational cost.

These studies support a multidimensional definition of patient facing quality. Accuracy and completeness remain necessary, but safe communication also requires readability, actionability, source traceability, uncertainty communication, cultural and educational fit, and evidence that users understand what the system has told them.

### Human factors, professional readiness, and multimodal expansion

Clinical implementation depends on professionals as well as models. Among Italian physiotherapists, Deodato et al. found widespread awareness of AI chatbots but limited clinical use: 66.9 percent had never used them in practice even though 78 percent expressed a positive attitude toward future adoption. Concerns centred on patient self diagnosis, false information, and loss of human interaction. In a sample drawn principally from Ecuador, Cherrez Ojeda et al. similarly found cautious optimism, but only 31.99 percent had used ChatGPT and 67.85 percent of familiar respondents regarded sole source clinical use as unethical. Safi et al. documented a different mismatch in surgical education: 80.9 percent had prior ChatGPT experience, yet 93.2 percent lacked formal AI training. Informal exposure is therefore advancing faster than structured professional education.

The multimodal papers widen the definition of clinical AI while also illustrating why adding data modalities should not be assumed to improve performance. Manir et al. combined textual embeddings with acoustic summaries for affective tone analysis in aphasia. Under recording separated evaluation, the fused model did not outperform the text only model, and human agreement on aphasic affect was low. Hussain et al. review evidence in depression detection showing benefits from combining EEG, speech, facial, and linguistic signals, but also report the familiar decline from controlled laboratory results to more ecologically representative datasets. Siefert et al. demonstrate that smaller fine tuned models can approximate expert psychological ratings of complex narratives, particularly when model outputs are ensembled, yet they retain human supervision as the appropriate clinical frame.

Multimodality also creates new risks. Jędrasiak and Bijoch examine synthetic audiovisual material in telemedicine and teledentistry, showing that manipulation can threaten diagnostic evidence, communication, and medico legal documentation. Their work makes provenance part of clinical safety. Trustworthy generative AI cannot be defined only by whether generated text is factually correct; healthcare systems must also be able to establish whether the data supplied to an AI system, or returned through a digital encounter, are authentic.

### Trustworthiness, governance, and accountable integration

The collection therefore shifts attention from model capability toward an evaluation and governance problem. Knott et al. show that healthcare AI studies still concentrate disproportionately on accuracy while paying far less attention to fairness, calibration, deployment readiness, and actual patient care. Their European analysis also makes clear that regulatory obligations depend on intended function and clinical risk. Administrative quality tools and systems that influence diagnosis or treatment cannot be governed as equivalent applications. Wang, Hao and Chen extend the accountability question into pharmaceutical intellectual property, where AI assisted discovery raises unresolved issues concerning inventorship, copyright, and ownership across jurisdictions.

Reproducibility deserves particular attention. A clinical system that changes a medically material recommendation when the patient information has not changed poses a different problem from a deterministic medical device, even if its average performance is favourable. The tumor board literature synthesized by Konzmann et al. reports substantial variation in repeated response consistency and incomplete reporting of model inference settings across studies. Oami et al. likewise show that repeated session consistency can deteriorate as extraction becomes more complex. Model updates introduce another layer of temporal instability because the product evaluated today may not be behaviourally identical to the product deployed later.

The evidence across the Research Topic supports a risk proportionate interpretation of human oversight. Lower consequence, reversible, and auditable tasks such as drafting, extraction, summarization, and guideline retrieval are plausible candidates for supervised implementation. Patient communication and guideline based decision support require stronger controls because deficiencies in readability, actionability, or omitted context may affect behaviour. Diagnosis, staging, treatment recommendation, and other high consequence applications require prospective validation, explicit uncertainty handling, and clinician accountability. Human review should therefore be designed into the workflow rather than appended as a generic disclaimer.

Figure 1 represents this translational logic. Foundation model capability is only the first stage. Task adaptation must be followed by evidence grounding, workflow integration, clinical validation, and continuous governance. Human oversight, external validation, reproducibility, evidence traceability, health literacy and equity, privacy, and accountability operate across the pathway rather than at a single endpoint. This structure also explains why no universally superior medical language model emerges from the collection. Different systems lead on different endpoints, and the preferred model changes with the task, population, infrastructure, privacy requirements, and tolerance for error.

**Figure 1:**
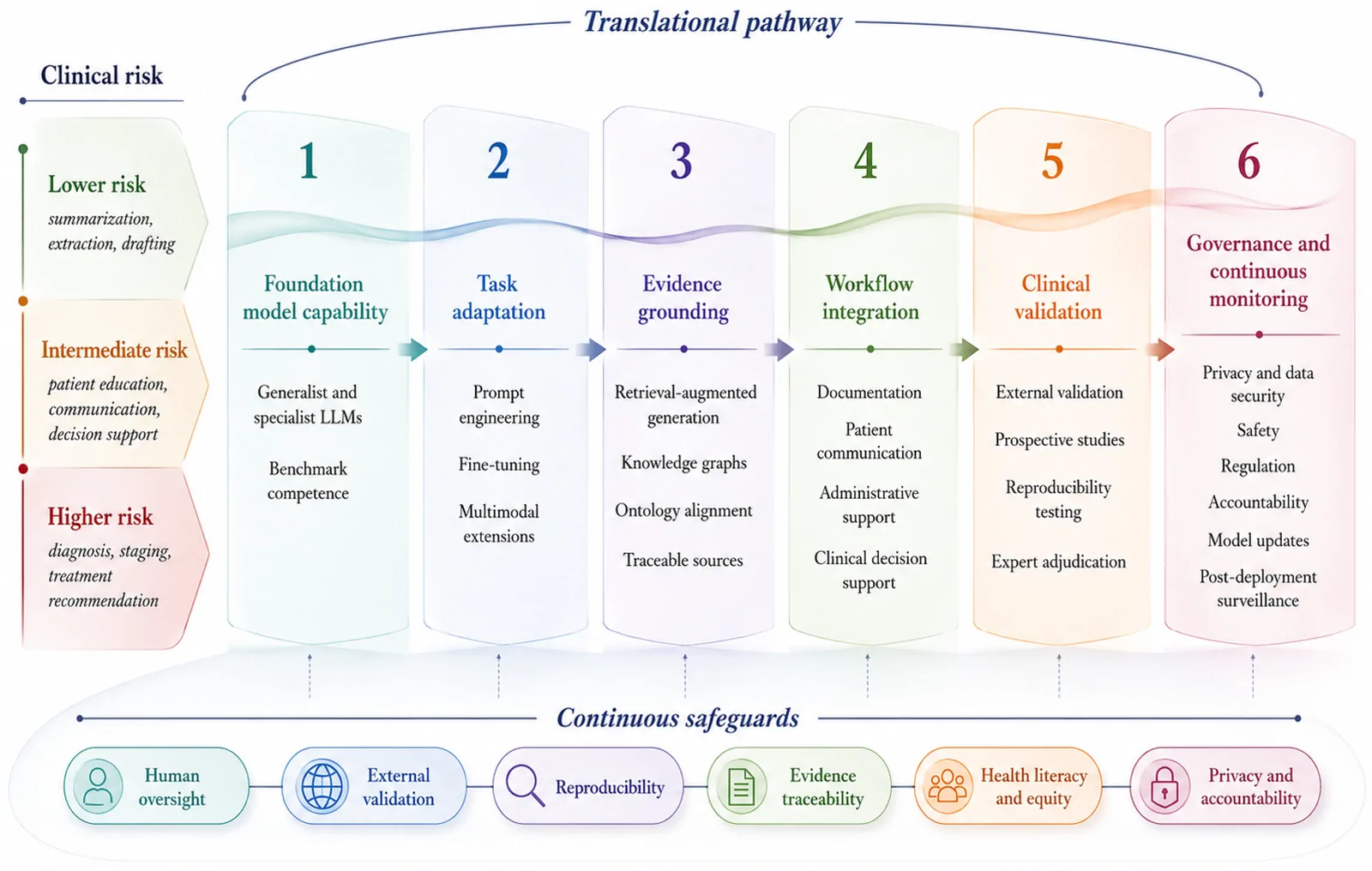
From generative AI capability to accountable clinical integration. The pathway links model capability to task adaptation, evidence grounding, workflow integration, clinical validation, and continuous governance. Safeguards operate across all stages and intensify as clinical risk increases.

## Conclusion

The 34 contributions document a field moving from proof of language competence toward the science of accountable clinical integration. Contemporary models can perform useful work in structured clinical reasoning, data extraction, patient education, documentation, knowledge retrieval, psychological assessment, and administrative processes. They can sometimes match or exceed junior clinicians or established information sources on narrowly defined measures. Those findings are scientifically relevant, but they are not equivalent to evidence of autonomous clinical readiness.

The next phase of research should therefore privilege prospective and multicentre evaluation, external validation across institutions and populations, repeated testing for response stability, explicit reporting of model versions and inference conditions, patient centred endpoints, economic evaluation, fairness assessment, and post deployment surveillance. Grounded architectures using curated retrieval, ontologies, knowledge graphs, local models, and explicit escalation rules warrant continued evaluation because they make model behaviour more constrained and auditable. Clinical AI literacy must develop in parallel so that professionals can judge uncertainty, verify sources, protect patient data, recognize inappropriate automation, and retain responsibility for consequential decisions.

Generative AI is becoming useful in medicine before it has become uniformly trustworthy. The evidence in this Research Topic suggests that the appropriate response is neither unrestricted adoption nor categorical rejection. The defensible path is selective integration in which technical capability is matched to clinical risk, evidence is made traceable, validation extends beyond benchmark accuracy, and accountability remains explicit throughout the system lifecycle.

